# Energy, Water and Fish: Biodiversity Impacts of Energy-Sector Water Demand in the United States Depend on Efficiency and Policy Measures

**DOI:** 10.1371/journal.pone.0050219

**Published:** 2012-11-21

**Authors:** Robert I. McDonald, Julian D. Olden, Jeffrey J. Opperman, William M. Miller, Joseph Fargione, Carmen Revenga, Jonathan V. Higgins, Jimmie Powell

**Affiliations:** 1 Worldwide Office, The Nature Conservancy, Arlington, Virginia, United States of America; 2 School of Aquatic & Fishery Sciences, University of Washington, Seattle, Washington, United States of America; 3 Freshwater Focal Area Program, The Nature Conservancy, Chargin Falls, Ohio, United States of America; 4 Department of Chemical and Biological Engineering, Northwestern University, Evanston, Illinois, United States of America; 5 North America Region, The Nature Conservancy, Minneapolis, Minnesota, United States of America; 6 Marine Focal Area Program, The Nature Conservancy, Arlington, Virginia, United States of America; 7 Freshwater Focal Area Program, The Nature Conservancy, Chicago, Illinois, United States of America; University of Nottingham, United Kingdom

## Abstract

Rising energy consumption in coming decades, combined with a changing energy mix, have the potential to increase the impact of energy sector water use on freshwater biodiversity. We forecast changes in future water use based on various energy scenarios and examine implications for freshwater ecosystems. Annual water withdrawn/manipulated would increase by 18–24%, going from 1,993,000–2,628,000 Mm^3^ in 2010 to 2,359,000–3,271,000 Mm^3^ in 2035 under the Reference Case of the Energy Information Administration (EIA). Water consumption would more rapidly increase by 26% due to increased biofuel production, going from 16,700–46,400 Mm^3^ consumption in 2010 to 21,000–58,400 Mm^3^ consumption in 2035. Regionally, water use in the Southwest and Southeast may increase, with anticipated decreases in water use in some areas of the Midwest and Northeast. Policies that promote energy efficiency or conservation in the electric sector would reduce water withdrawn/manipulated by 27–36 m^3^GJ^−1^ (0.1–0.5 m^3^GJ^−1^ consumption), while such policies in the liquid fuel sector would reduce withdrawal/manipulation by 0.4–0.7 m^3^GJ^−1^ (0.2–0.3 m^3^GJ^−1^ consumption). The greatest energy sector withdrawal/manipulation are for hydropower and thermoelectric cooling, although potential new EPA rules that would require recirculating cooling for thermoelectric plants would reduce withdrawal/manipulation by 441,000 Mm^3^ (20,300 Mm^3^ consumption). The greatest consumptive energy sector use is evaporation from hydroelectric reservoirs, followed by irrigation water for biofuel feedstocks and water used for electricity generation from coal. Historical water use by the energy sector is related to patterns of fish species endangerment, where water resource regions with a greater fraction of available surface water withdrawn by hydropower or consumed by the energy sector correlated with higher probabilities of imperilment. Since future increases in energy-sector surface water use will occur in areas of high fish endemism (e.g., Southeast), additional management and policy actions will be needed to minimize further species imperilment.

## Introduction

In the United States (US), the energy sector is responsible for more than half of all water withdrawals [1]. Only a fraction of this water is consumed, with the remainder returned to the hydrologic system after likely modification of its physical (e.g., flow regimes, temperature) and chemical (e.g., dissolved oxygen) properties. With continued population growth and economic development in the US, total energy consumption is expected to increase over the coming decades. At the same time, the combination of energy sources used by Americans is changing, driven by shifts in the availability, cost effectiveness, and investment in emerging and traditional technologies. For example, new techniques for extracting natural gas from shale have increased supply and decreased the price of natural gas, affecting investments in new production in many other energy technologies, as well as increasing the water used for extracting natural gas [2], [3]. Moreover, concerns about energy security and the environmental impacts of energy production are leading policymakers to change the incentives and regulations that govern the energy sector. For instance, the US has made significant investments in subsidizing biofuel production, incentivizing new renewable electric generation capacity, and funding research into developing commercially viable technologies for carbon capture and storage (CCS) of emissions from fossil fuels, particularly coal [4]. Another example is potential new EPA regulations under Section 316(b) of the Clean Water Act that may force some thermoelectric plants to switch from current once-through cooling to recirculating cooling. Section 316(b) requires that facilities use the best available cooling technology to minimize environmental impacts, which in some cases may require facilities that currently use once-through cooling to transition to using recirculating cooling.

The future expansion of energy consumption and changes in the use of different sources could cause major changes in energy sector water use. Water withdrawal and consumption by the energy sector may increase in some areas [5], altering water quality and quantity in freshwater ecosystems [6], [7], and further threatening an already imperiled fauna. In the US, freshwater taxa already have a greater proportion of their species imperiled than terrestrial taxa [8], and are expected to disappear at a rate five times that of terrestrial fauna in the future [9]. The potential for future energy sector withdrawals to worsen the current freshwater biodiversity crisis appears high, but remains uncertain. The central goal of this paper is to explore the relationships between energy policies on water use and the implications of these energy-related water impacts on freshwater ecosystems, specifically freshwater fishes. Freshwater fishes are a useful indicator taxa for freshwater biodiversity more broadly: they are widely distributed across the US, their abundance and distribution patterns commonly reflect impacts to many other components of freshwater biodiversity [10], they respond to changes in water consumption and withdrawal [11], and they contribute valuable goods and services to human society [12].

**Figure 1:**
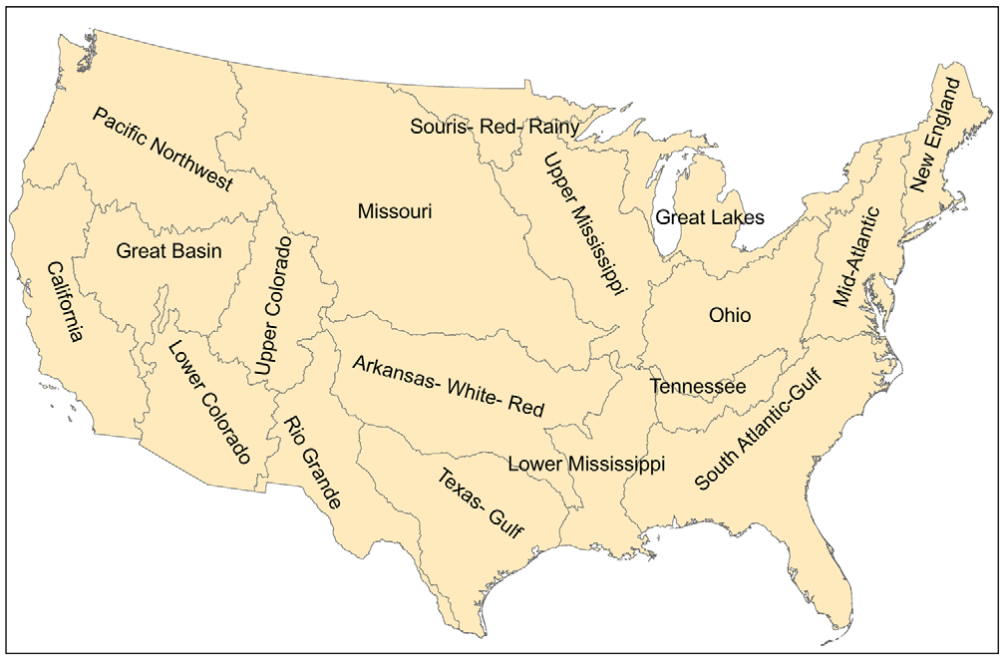
Water resource regions. The 18 water resource regions of the United States, as defined by the 2-digit Hydrologic Unit Codes (HUC) of the USGS.

A number of recent studies have looked at how changes in the energy sector will affect water withdrawals or consumption [5], [13]–[24]. In this paper, we have the following objectives:

1. Synthesize information on the water-use intensity (m^3^GJ^−1^) of various energy production techniques, using high and low values of water-use intensity to provide a realistic range for each technique;
2. Present scenarios of future water withdrawal and consumption by the energy sector; and
3. Compare energy sector water-use with current patterns of threats to endangered fish species by major water resource region (Figure 1).

We present three scenarios of water use, based upon energy production scenarios developed by the US Energy Information Administration (EIA).

- **Reference Case**- The “business as usual” scenario, depicting the future of US energy markets under current policies, with baseline assumptions about the rate of economic growth, world oil price, and the development of new technologies.
- **Greenhouse Gas (GHG) Price Case**- Similar to the Reference Case scenario, but a price is set on greenhouse gas emissions throughout the entire US economy.
- **Extended Policies Case**- Similar to the Reference Case scenario, but additional government regulations foster improved electricity and liquid fuel efficiency.

## Materials and Methods

We first estimated water-use intensity for energy production techniques, drawing heavily from several of the published reviews of this topic [17]–[19], [22]–[24]. We then obtained scenarios of future energy demand and multiplied those by water-use intensity to estimate total water use. We partitioned the new energy production and water use among major water resource regions, using geospatial information on the supply of needed natural resources and demand for energy. Finally, we statistically compared the water withdrawal and consumption by energy type in each of the water resource regions with the probability of a fish species being imperiled, and then used this statistical model to estimate the likely impact future energy sector water use would have on freshwater fishes as an indicator of freshwater biodiversity more broadly.

### Calculating water requirements

We recognized 12 energy production techniques (solar photovoltaic, solar thermal, wind, geothermal, biopower, hydropower, coal, nuclear, natural gas, power generated from municipal waste, biofuels, and petroleum), based upon the sectors used in the Annual Energy Outlook [4]. Except where noted, we define each energy production technique identically in scope to the Annual Energy Outlook. For certain energy types, we recognize multiple different techniques for producing energy that have very different water footprints (e.g., a coal power plant that is once-through cooled versus one that uses closed-loop cooling).

In this paper, we define “water use” as any use of surface water or groundwater to produce energy, including water used for hydropower production and water used to irrigate bioenergy feedstocks. Note that other papers have categorized water use into three categories: “blue”, “green ,” and “gray”[25]. Our definition of “water use” is roughly analogous to “blue water,” defined as the amount of water taken from groundwater or surface water that does not return to the catchment from which it is withdrawn. We do not estimate “green” water use (i.e., the amount of rainwater used by crops) or “grey” water use (i.e., the amount of water needed to safely dilute pollutants or impurities) (cf. [14], [15], [16]).

We divide “water use” into two subcategories: water withdrawn/manipulated, the removal of water from a surface or groundwater source; and water consumption, the portion of water withdrawal that is not returned to the environment but is consumed by the process of energy production. This consumption can take several forms, including evaporation (e.g., in the cooling loop of a thermoelectric plant or from a reservoir), transpiration (e.g., irrigation water applied to energy crops), or incorporation into a product, byproduct or material of production (e.g., water used in biofuel production). Note that our definition of “withdrawal/manipulation” includes water that is removed from a river system only briefly, as water passed through a hydropower turbine located at a dam site. By classifying water used by hydropower as water manipulated, we are using a terminology that differs from the United States Geological Survey, which presents statistics on hydropower water use separate from water use for thermoelectric plant cooling. We adopted this different terminology because one of our primary goals in this paper is to present a full picture of the water used for energy production, including how that water use affects freshwater ecosystems. Dams can affect a river's flow regime, connectivity and water quality and are among of the leading sources of threat for aquatic species. Therefore, quantifying the volume of water that is run through hydropower dams' turbines (i.e., manipulated) provides relevant information on how water management by hydropower affects fish species.

We recognize two major parts of the production process in our estimate of water-use intensity (m^3^ of water per GJ of energy): material/resource acquisition and processing (e.g., mining coal and preparing it for use) (Table 1); and electricity generation (Table 2). Note that the endpoint of our production analysis is the delivered energy embodied in electricity or liquid fuels. We do not account for energy that is lost during energy consumption, for example, when electricity is converted to heat for a home or when liquid fuels are converted to a car's kinetic energy. Similarly, the beginning of our analysis of the production process is material/resource acquisition and processing. We do not account for water or energy, for example, used to create the steel in the machinery that mines the coal.

**Table 1:** Statistics for material resources acquisition/processing.

Type	Withdrawal/manipulation (m^3^GJ^−1^)		Consumption (m^3^GJ^−1^)		Water-intensity varies by	EIA forecasts by	Notes
	*Low*	*High*	*Low*	*High*			
Solar PV	0.486	0.549	0.061	0.161	National	Elec. Producing regions	High withdrawal value: 60% mono-SI, 40% multi-SI. Proportional split based on [42]. Low withdrawal value: 48% mono-SI, 32% multi-SI, 20% CdTe representing hypothetical future split. Water use values from [23]. Consumption: High and low values from [43].
Solar Thermal	0.0825	0.36	0.024	0.105	National	Elec. Producing regions	High values: 0.105 m^3^GJ^−1^ consumption for plant construction and O&M [43]. Low values: 0.024 m^3^GJ^−1^ consumption for plant construction and O&M [43]. Adjustment for withdrawal based on observed rates for solar power [43] and the ratios between withdrawal and consumption for construction shown above for the high water use number for solar PV.
Wind	0.047	0.089	0.011	0.019	National	Elec. Producing regions	Withdrawal: Taken from [23]. High value is Denmark highest example, low value is Denmark lowest example. Consumption: Taken from [44],Table 6.
Geothermal	0.003	0.031	0.001	0.011	National	Elec. Producing regions	Consumption data from Table 4-3 in [45]. Withdrawals assumed 3x bigger.
Coal	0.028	1.21	0.003	0.328	National	Coal producing regions	Withdrawal: [23], low number is for western surface mining and train transportation, high number is for eastern underground mining and slurry pipeline. Withdrawal: [23], low number is for surface mining and train transportation, high number is for underground mining and slurry pipeline.
Nuclear	0.083	0.392	0.047	0.159	National	Elec. Producing regions	Withdrawal: [23], low number is for centrifuge enrichment, top number is for diffusion enrichment. Consumption: Range taken from [17]. Brackets other studies reported values. There is variation by mining type and method of enrichment, which is captured within this range.
Natural Gas	0.033	0.153	0.025	0.036	National	Natural gas producing regions	Withdrawal: [23], low number for offshore extraction, high number on-shore (plus other components). Consumption: Low value from [23], high number assumed 50% above their number. Interestingly, our high estimates are above some estimates of the extra use for shale gas- like [2] estimated 5–29 gallons per MWh, which gives 0.0189–0.110 m^3^GJ^-1^.
Hydropower	0.00	0.00	0.00	0.00	National	Elec. Producing regions	Dam construction assumed trivial relative to water use for electricity production.
Municipal Waste	0.00	0.00	0.00	0.00	National	Elec. Producing regions	Assumed municipal waste streams would have been created anyway, so no water for waste creation.
Petroleum	0.22	0.27	0.07	0.21	National	Petroleum producing regions	[46] lists average injection water withdrawal as 8 gal H20/gal oil, with consumption varying from 2.1-5.4. Processing can vary from 0.5-2.5, and we assume all processing withdrawals are consumed.
Biopower	0.00	0.00	0.00	0.00	National except for energy crops, which are state-level	Biomass market	Assumed zero except for energy crops, because the waste would have been collected and stored anyway. Assumed all rain-fed for energy crop biomass market.
Biofuel- corn	19.4	24.3	16.4	19.7	State-level [47]	Biomass market	High number is existing average, averaging across irrigated and non-irrigated acres. Low number is estimated value for 2035, with an increase in yield and a full-switch to pressure irrigation (and thus no gravity irrigation).
Biofuel- soybean	58.3	71.7	49.6	53.6	State-level [47]	Biomass market	High number is existing average, averaging across irrigated and non-irrigated acres. Low number is estimated value for 2035, with an increase in yield and a full-switch to pressure irrigation (and thus no gravity irrigation).
Biofuel- cellulosic	0.0	0.0	0.0	0.0	State-level [47]	Biomass market	Assumed all rain-fed. See text for details.

**Table 2:** Statistics for electricity generation.

Type	Withdrawal/manipulation (m^3^GJ^−1^)		Consumption (m^3^GJ^−1^)		Water-intensity varies by	EIA forecasts by	Notes
	*Low*	*High*	*Low*	*High*			
Solar PV	0.004	0.021	0.004	0.021	National	Elec. Producing regions	[43] and [23]
Solar Thermal	0.58	1.06	0.58	1.06	National	Elec. Producing regions	Withdrawal and consumption: range in [23] of commercial operational technologies (excluding dish sterling and dry cooling).
Wind	0	0.001	0	0.001	National	Elec. Producing regions	[23]. Assumed all water consumed.
Geothermal	1.89	12.4	0.66	1.89	Varies by mix of open and recirculating cool in each elec. Producing region	Elec. Producing regions	[23]
Coal: once-through cooling	21.1	52.6	0.35	1.23	Varies by mix of open and recirculating cool in each elec. Producing region	Elec. Producing regions	Withdrawal: [17]. Consumption: Lower is [17] example of open loop, upper is highest value in [23].
Coal: recirculating cooling	0.35	1.23	0.31	1.23	Varies by mix of open and recirculating cool in each elec. Producing region	Elec. Producing regions	Withdrawal: lower is from closed loop tower example in [17], upper is from wet tower, subcritical example in [23]. Consumption: Lower is [17] example of IGCC, dry fed with cooling tower, upper is highest value in [17].
Nuclear: once-through cooling	26.3	63.1	0.42	0.94	Varies by mix of open and recirculating cool in each elec. Producing region	Elec. Producing regions	Withdrawal: [17]. Numerous other sources fell in this range. Consumption: Lower value from [48]. Upper value from maximum consumption of any plant in [23]. Note that some nuclear power plants use saline or brackish water for cooling, which may inflate average water-use statistics cited in the literature.
Nuclear: recirculating cooling	0.59	1.19	0.45	0.94			Withdrawal: [17]. Numerous other sources fell in this range. Consumption: Lower value from [17]. Upper value from maximum consumption of any plant in [23].
Natural Gas: once-through cooling	7.89	52.6	0.11	0.35	Varies by mix of open and recirculating cool in each elec. Producing region	Elec. Producing regions	Withdrawal:[17], low end natural gas CC with open loop, high end [17] for generic steam plant, open loop. Consumption: low end [17] for NGCC open loop, high end [17] for generic steam plant, open loop.
Natural Gas: recirculating cooling	0.25	0.66	0.20	0.54			Withdrawal: Low value from [17], NGCC closed loop tower, upper value from [17] closed loop generic steam plant. Consumption: low from [17], NGCC, closed loop, upper value from [17]closed loop generic steam plant.
Hydropower	1,811 (US mean)	2,173 (US mean)	4.6 (US mean)	14.1 (US mean)	State-level	Elec. Producing regions	Manipulation: Calculated from head of dams listed in the National Inventory of Dams [36]. For average head in each electricity producing region, high number is 75% turbine efficiency, low number is 90% turbine efficiency. Consumption: NREL data [49].
Municipal Waste	6.6	16.7	0.1	0.5	National	Elec. Producing regions	[40] generic thermoelectric plant numbers
Petroleum: once-through cooling	21.1	52.6	0.35	0.52	Varies by mix of open and recirculating cool in each elec. Producing region	Elec. Producing regions	Withdrawal: [17], generic open-loop thermoelectric plant. Consumption: lower [17], generic open-loop thermoelectric plant. Upper assumed 50% higher.
Petroleum: recirculating cooling	0.35	0.66	0.35	0.52			Withdrawal: [17] generic closed loop thermoelectric (tower). Consumption: [17] generic closed loop thermoelectric plant.
Biopower	6.6	16.7	0.1	0.5	Varies by mix of open and recirculating cool in each elec. Producing region	Elec. Producing regions	[23] generic thermoelectric plant numbers

For each part of the production process, we defined a high- and low-end estimate of water-use intensity (for both withdrawal/manipulation and consumption). Generally, the high-end estimate gives current levels of water-use intensity as reported in the literature, whereas the low-end estimate of water-use intensity is the lowest level reported in the literature or the lowest level likely with future technological changes (cf. [26]). This approach with a high and low estimate is meant to represent the uncertainty about future water-use intensity. It does not consider the potential impact of catastrophic events, such as an oil spill, on water resources.

There is a large variation in water-use intensity, in terms of withdrawal/manipulation, between thermoelectric plants that use once-through cooling and those that use recirculating cooling (Table 1). To reflect this fact, we have calculated at a regional level the proportion of plants that use these various technologies, using a comprehensive commercial database of the plants within the US created by the Ventyx Corporation [27]. The Ventyx data is useful because it has the spatial locations of all significant electricity producing facilities in the United States, allowing us to accurately determine what watershed the facility is located in. Our high-end estimates for 2035 assume that all existing plants continue with current cooling technology, but that all new plants use recirculating cooling technology, consistent with the general trend for recirculating cooling plants to become a greater proportion of the nation's thermoelectric plants. Our low-end estimates for 2035 assume that, between 2010 and 2030, all existing plants that use once-through cooling are converted to recirculating cooling systems. Such a transition would be expensive, and would likely only come about through some regulatory requirement, such as changes to regulations developed by the EPA around Section 316(b) of the Clean Water Act. It is important to note that existing regulations around Section 316(b) are unlikely to require such a large change in cooling technologies, and that such a change is not necessarily cost-effective or beneficial to biodiversity in all situations. However, we have included this scenario in our low-end estimates for 2035 to show the potential water withdrawal/manipulation reduction if existing plants are slowly transitioned to recirculating cooling systems.

We have included water used for the irrigation of bioenergy feedstocks in our calculations of material acquisition for biopower and biofuel production. There are three major feedstocks considered in our analysis: corn, soybean, and cellulosic.

Corn and soybean are the two feedstocks currently used for commercial biofuel production, and for these two our high-end estimate is simply a function of the current average state-level irrigation rate of the crops (m^3^/tonne). The advantage of using state-level estimates is that it accounts for the considerable variation in irrigation rates between corn raised in, for instance, Indiana (primarily rain-fed) and Nebraska (a significant portion irrigated). Our low-end estimate for corn and soybean in 2035 assumes continued gains in the yield of these crops with no additional inputs of water required, consistent with historical trends, plus a transition away from gravity-fed irrigation toward more efficient sprinkler systems, also consistent with historical trends. We note that rainfed biofuel crops also transpire significant amounts of water [28], which could otherwise be used for other purposes, such as food production; however, this “green water” use is beyond the scope of our paper.

The third major feedstock we consider is generic biomass used for either cellulosic ethanol or for biopower.Here, we recognized five sources of biomass, consistent with NREL research into price-supply curves for each of these sources [29]: urban waste wood; mill waste wood; forestry residuals; agricultural residues, and dedicated biomass crops. All are assumed to have no additional water-use involved with their creation and use for energy purposes. For instance, forestry residues would exist anyway without a market for biomass, and so it is assumed that there is no extra “blue” water involved with their use in bioenergy projects. For dedicated biomass crops, we assume there is no irrigation involved in production (i.e., the crops are all rain-fed). This assumption is consistent with that adopted in the US Department of Energy's Billion-Ton Update study [30], and seems plausible given that the relatively low profit margins considered likely for cellulosic feedstock production may make intensive production with irrigation cost prohibitive.

Finally, to estimate the amount of water saved when a unit of energy is not consumed, due to either efficiency gains or reductions in demand, we calculated the average water withdrawal/manipulation and water consumption per unit energy for both the liquid fuel sector and for the electricity sector. To derive these values, we first calculated total energy consumption for each sector in 2010 and divided by the total water use for each sector.

### Scenarios of future energy use

Our energy scenarios are taken from the EIA's Annual Energy Outlook (AEO) 2011 [4]. These scenarios were calculated by EIA's National Energy Modeling System, a comprehensive econometric model of US energy production, imports, and consumption. For each energy production technology, each EIA scenario projects energy produced and (for electricity) generation capacity, from now until 2035, by subregion. For electricity-producing technologies, 22 electric market subregions, whose boundaries delineate areas of the US electric grid that are relatively disconnected from one another, are used. Additionally, projections of old plant capacity that will be retired and new plant capacity that will be created are available. For coal, oil, and natural gas production, resource extraction is also listed by major geographic regions of the US. For each technology, we applied our high and low water-use intensity estimates for each subregion, taking into account that the material/acquisition and processing component and the electric generation component may occur in different subregions.

We present three water use scenarios, based upon energy production scenarios developed by the EIA. Much more information on these standards available in the AEO [4].

- **Reference Case**- This scenario incorporates baseline economic growth (2.7% per year economic growth between 2009 and 2035), increasing crude oil prices (rising to $125 per barrel), and assumes that the Renewable Fuel Standard target will be met in the immediate future.
- **Greenhouse Gas (GHG) Price Case**- Similar to the Reference Case scenario but it applies a price for CO_2_ emissions throughout the economy. The CO_2_ price starts at $25 per ton beginning in 2013 and increases to $75 per ton by 2035.
- **Extended Policies Case**- This scenario differs from the Reference Case in that additional government regulations foster improved electricity and liquid fuel efficiency. It assumes new light duty vehicle CAFE standards (to 46 miles per gallon by 2025) and tailpipe emissions standards, and includes additional rounds of efficiency standards for currently covered products, as well as new standards for products not yet covered.

### Where energy sector water impact occurs

The next phase of the analysis involved apportioning the water use predictions by various subregions among 18 major water resource regions for the contiguous US, as defined by the USGS Hydrologic Unit Code (HUC) system. Our general strategy was to use higher-resolution information on where material acquisition/resource processing or electricity generation occurs to partition the water use as accurately as possible. For projections into the future, we used information on both current and proposed energy facilities contained in the Ventyx database to partition the water use.

For material acquisition/resource processing, the method used varied by energy technique (Table 1). For technologies that involved resource extraction (coal, natural gas, petroleum, uranium), we used maps of production areas available from the EIA. Bioenergy irrigation was partitioned using a map of irrigated area in the US [31]. Biofuel processing was partitioned using information on the location of current and proposed biofuel production facilities from the Ventyx database. For other energy production techniques, material acquisition/resource processing is small relative to water use for electricity generation, and it was partitioned proportional to electric generation (see below), essentially assuming that most material acquisition/resource processing occurs in the same major water resource regions where this electricity is generated.

For electricity generation, we used the Ventyx database to calculate, for each energy production technique, the total electricity generation in each major water resource region. Since the location, as well as the capacity (MW), of most facilities is known with great precision, it is possible to calculate this accurately. Water-use was then partitioned among major water resource regions using this calculation.

### Energy sector water use and freshwater biodiversity

Data on the status, source of imperilment, and geographic range of US freshwater fish species were taken from NatureServe [32], as updated in Mims et al. [33]. Fish species were classified as imperiled according to NatureServe conservation ranking categories of G1 (defined as at very high risk of extinction due to extreme rarity, such as 5 or fewer populations, very steep declines, or other factors) and G2 (defined as at high risk of extinction or elimination due to very restricted range, very few populations, steep declines, or other factors) [34]. Next, for all 239 imperiled freshwater fish species detailed text descriptions of the source of imperilment were used to assign threats to species from 9 major threat categories: dams/impoundments; invasive/introduced species; altered hydrologic flow/channelization; overharvesting/overfishing; pollution/water quality; sedimentation/turbidity/siltation; excess water consumption/withdrawal; and hybridization. Most species had more than one threat listed, with dams/impoundments being most commonly listed (36.0% of species), followed by pollution/water quality threat (32.6% of species).

While water used in cooling of thermoelectric power plants is overwhelmingly from surface water, water used for irrigation is frequently obtained from groundwater [1]. The effects of groundwater use on freshwater fish species are often different than those of surface water use. Although there are linkages between groundwater use and the quantity and quality of surface water, these links are complex and depend on the hydrology of the river basin and underlying aquifer. In order to make our estimates of water use more biologically meaningful, we used county-level information on the proportion of withdrawals from groundwater and surface water to split water use into its surface and groundwater components. For this calculation, all thermoelectric cooling water use was split into surface and groundwater components based upon the proportion of freshwater withdrawals from groundwater reported for the power sector, while irrigation water use was split into surface and groundwater components based upon the proportion of freshwater withdrawals reported from groundwater for the agriculture sector [1]. For our statistical analysis, we have only used information on the surface component of withdrawal or consumption, since the amount of groundwater used by the energy sector is a relatively small portion of the total groundwater withdrawal in most basins and hence seemed unlikely to be statistically related to fish imperilment. Moreover, the amount of water available in groundwater basins is often unknown or poorly characterized, making the normalization (see below) of groundwater use difficult.

**Table 3:** Average water availability by major hydrologic region.

Water Resource Region	Average flow, million m^3^/yr (1901–2009)
New England	97,100
Mid-Atlantic	133,600
South Atlantic-Gulf	306,400
Great Lakes	143,200
Ohio	188,900
Tennessee	58,000
Upper Mississippi	96,700
Lower Mississippi	111,300
Souris-Red-Rainy	8,100
Missouri	76,900
Arkansas-White-Red	41,100
Texas-Gulf	24,100
Rio Grande	6,200
Upper Colorado	16,700
Lower Colorado	5,600
Great Basin	15,600
Pacific Northwest	223,700
California	117,500

Next, we normalized surface water use by available water, to obtain the proportion of water used for energy production in each subregion. Specifically, we divided three metrics of water-use (hydropower water manipulation, non-hydropower energy sector surface water withdrawals, and energy sector surface water consumption) by average annual surface water availability in each water resource region [35] (Table 3). Hydropower water manipulation was treated separately from non-hydropower energy sector surface water withdrawal for this calculation to see if there were different patterns for the two subcomponents of withdrawal/manipulation. Finally, we calculated the range-area-weighted average of normalized water use for each fish species:

where *R_i_* is the total range size (area) of the species in major hydrologic region *i,* and *U_i_* is the normalized water use in major hydrologic region *i*.

We tested two related hypotheses using logistic regression analysis. First, we tested to see if expert evaluation of the threats facing each species is consistent with our metrics of water use, simply examining whether fish species with a particular reported threat had higher relative water-use on the appropriate metric (e.g, species threatened by dams and hydropower water use). Second, we tested to see if the probability of fish species imperilment is positively correlated with one of our three metrics of water-use (hydropower water manipulation, non-hydropower energy sector surface water withdrawals, and energy sector surface water consumption), after accounting for species range size. For each logistic regression analysis, we first added the term for species range, then the term for normalized water use, and then tested for any interaction terms. At each step, the significance of each addition was tested using likelihood ratio tests. When comparing between models using different metrics of normalized water use (i.e., not nested models), we used Akaike's Information Criterion (AIC). To improve normality of variables and meet the assumptions of logistic regression, our metrics of normalized water-use and species area were log-transformed.

## Results

### Energy production and consumption

**Figure 2:**
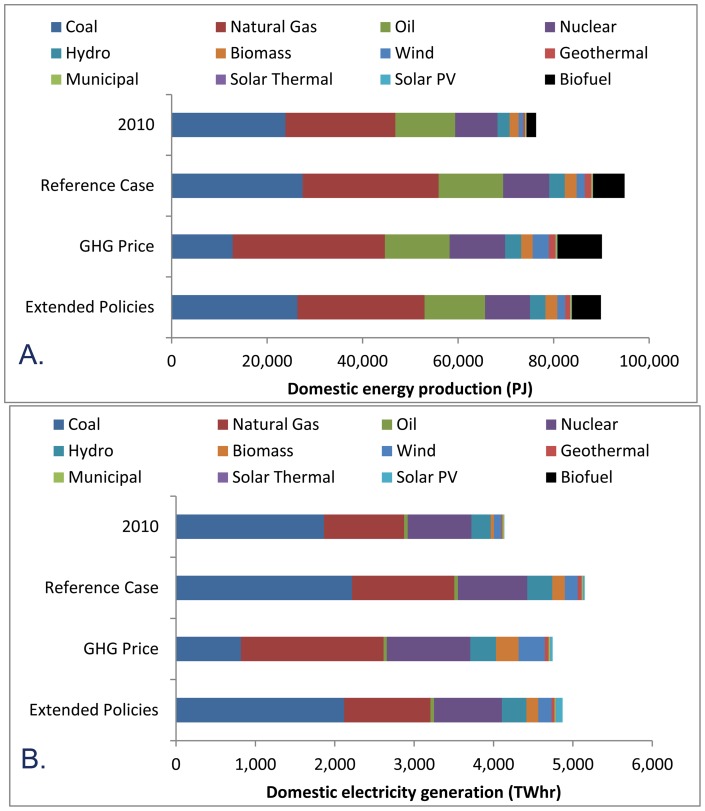
United States domestic energy creation. US annual energy production (A) and electricity generation (B), in 2010 and in 2035 for three scenarios of future energy policy. Annual energy production is shown in petajoules and electricity generation is shown in terawatt-hours.

Current domestic energy production is dominated by coal and natural gas [4], with significant contributions from oil production and nuclear energy (Figure 2A). By 2035, the Reference Case predicts an increase in production from all sources, particularly natural gas. There would also be a large increase in biofuel production, driven by US federal policy. The GHG Price Case predicts much less production from coal, and an increase in wind and biofuels, relative to the Reference Case. The Extended Policies Case predicts a similar combination of energy sources as the Reference Case, but with less overall production, as increased energy efficiency would reduce aggregate demand for energy.

Coal, natural gas, and nuclear dominate current domestic electricity generation (Figure 2B). According to projections under the Reference Case, electricity generation increases in most sectors, including the renewable technologies of wind and biomass. The Extended Policies Case includes a composition of energy production sources similar to the Reference Case, but with less total domestic electricity production due to the decreased demand from efficiency measures. The GHG Price Case predicts reduced electricity generation from coal and greatly increased generation from wind and biomass relative to the Reference Case.

### Water use intensity

**Figure 3:**
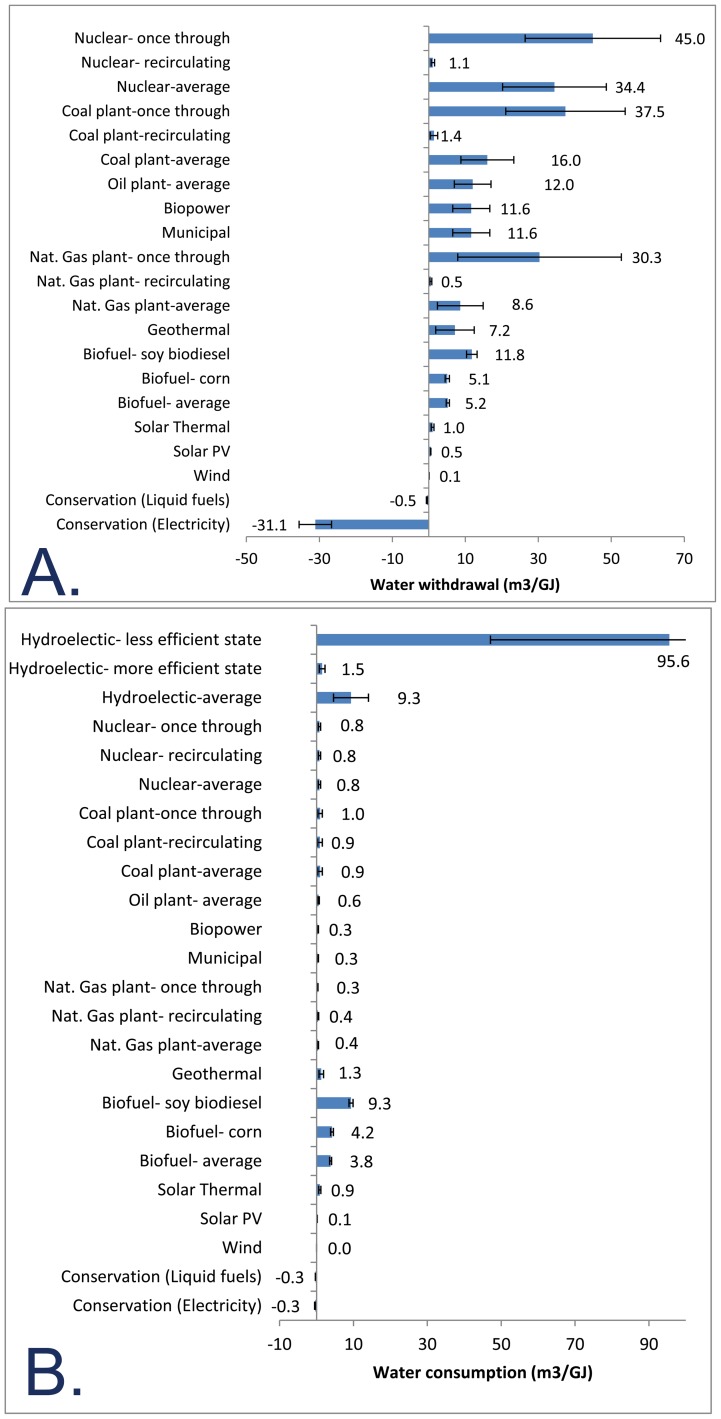
Water-use intensity of energy technologies. Water-use intensity (m^3^GJ^−1^) of US domestic energy production or energy conservation, in terms of water withdrawal (A) or water consumption (B). These water-use intensity estimates include water for material acquisition and processing, as well as for electricity generation where applicable. Errors bars indicate the range of our low and high water-use intensity estimates. The value labeled is the midpoint between these high and low estimates. The effect of energy conservation is shown using the energy mix in 2010. For hydropower, for display purposes typical consumption values are shown for more efficient and less efficient regions. Because hydropower water manipulation is more than an order of magnitude greater than water withdrawals for other technologies, hydropower is omitted in the top panel (A).

The water withdrawal/manipulation intensity of energy production techniques can vary over five orders of magnitude (Figure 3A). The largest water withdrawal/manipulation intensity is for hydropower. The national average water withdrawal/manipulation intensity for hydropower, weighted by electricity-production, is estimated as 1,810–2,170 m^3^GJ^−1^ (ranges for hydropower indicate uncertainty about the efficiency of turbines). However, the water withdrawal/manipulation intensity for hydropower varies greatly for different electricity producing regions. This large regional variation is primarily due to variation in hydrologic head [36]: electricity producing regions with dams with a large hydrologic head will have relatively smaller water withdrawal/manipulation intensities. For instance, the “Western Electricity Coordinating Council/Southwest” electricity producing region, which includes most of Arizona and New Mexico, has a large weighted average head, driven by big dams like the Hoover Dam and Glen Canyon Dam, and thus has a low estimated water withdrawal/manipulation intensity of 544–652 m^3^GJ^−1^. By contrast, the “Florida Reliability Coordinating Council” region has a small weighted average head, driven by the lack of topographic relief in Florida, and thus has a high estimated water withdrawal/manipulation intensity of 14,900–17,900 m^3^GJ^−1^.

For thermoelectric power production (e.g., from coal, natural gas and nuclear energy sources), the major difference in water withdrawal/manipulation intensity is between once-through cooling (high water withdrawal/manipulation intensity) and recirculating cooling (low water withdrawal/manipulation intensity). Nuclear power has higher average water withdrawal/manipulation intensity because a higher proportion of nuclear plants are once-through cooling than other types of thermoelectric plants. Natural gas power has lower average water withdrawal/manipulation intensity because combined cycle gas turbine plants (the dominant natural gas power plant type) generally use less cooling water. Finally, renewable energy production technologies, such as solar and wind, have among the lowest water withdrawal/manipulation intensities of the technologies assessed.

Energy conservation (reduced energy consumption caused by increases in energy efficiency or reduced demand) would reduce US water withdrawal/manipulation. This effect is greatest for the electric sector, where every 1 GJ of electricity conserved would save 27–36 m^3^ of water withdrawal/manipulation (midpoint of range, 31.1 m^3^GJ^−1^). By contrast, the effect would be less for the liquid fuel sector (0.4–0.7 m^3^GJ^−1^ of liquid fuels saved, midpoint estimate 0.5 m^3^GJ^−1^). This results in part because so much of the energy in the US liquid fuel sector comes from petroleum, which is extracted abroad and hence does not figure into the calculations of US water withdrawal/manipulation. Another reason for this trend is that the use of cooling water in many thermoelectric plants is so high relative to the water used in the extraction of petroleum.

Water-consumption intensity trends among sectors have somewhat similar patterns to those for water withdrawal/manipulation intensity (Figure 3B). Hydroelectric power demonstrates among the highest water consumption intensities due primarily to evaporation from reservoirs. The weighted-average water consumption intensity for the US is 4.6–14.1 m^3^GJ^−1^ (midpoint estimate 9.3 m^3^GJ^−1^). Note that some hydropower dams, including many of the largest, are multipurpose dams that also provide water supply or flood control benefits. Moreover, the amount of water evaporated off reservoirs per unit power produced varies among electricity producing region, depending on the climate (arid climates have more evaporation than humid climates) and the configuration of reservoirs (wide shallow reservoirs have more evaporation than narrow deep reservoirs). For instance, the “Texas Regional Entity” electricity producing region, which includes most of south Texas, has the largest estimated water consumption intensity of 47–144 m^3^GJ^−1^ (midpoint estimate 96 m^3^GJ^−1^), driven by a dry climate and relatively little topographic relief, which implies wide flat reservoirs. By contrast, the “Midwest Reliability Council -West” region, which includes places like Minnesota, has the lowest estimated water-consumption intensity of 0.7–2.3 m^3^GJ^−1^ (midpoint estimate 1.5 m^3^GJ^−1^), presumably because of the low evaporation rates off reservoirs in this relatively cold and humid climate.

Biofuel production also has high water consumption intensities, due to the high fraction of irrigation water that is either lost to evapotranspiration or incorporated into plant biomass. Compared with the large differences in water withdrawal/manipulation intensities, there is little difference in water consumption intensities between once-through and recirculating cooling thermoelectric plants. The large differences in the intensity of water withdrawal/manipulation are offset because the vast majority of water used in once-through cooling is returned rather than consumed. Geothermal and solar thermal have similar water consumption intensities to fossil fuel technologies. However, solar PV and wind have much lower water consumption intensity.

Energy conservation would also reduce US water consumption. This effect would be similar in size for the liquid fuel sector, where every 1 GJ conserved would save 0.2–0.3 m^3^ of water consumption (midpoint of range, 0.25 m^3^GJ^−1^) and for the electricity sector (0.1–0.5 m^3^GJ^−1^ of electricity saved, midpoint estimate 0.3 m^3^GJ^−1^). The effect of energy conservation of liquid fuels on US water consumption is high, relative to the situation with US water withdrawal/manipulation, because a fraction of liquid fuels come from biofuels, and irrigation water used for biofuel production has a much larger consumption of water per unit energy than other energy production techniques.

### Energy sector water use

**Figure 4:**
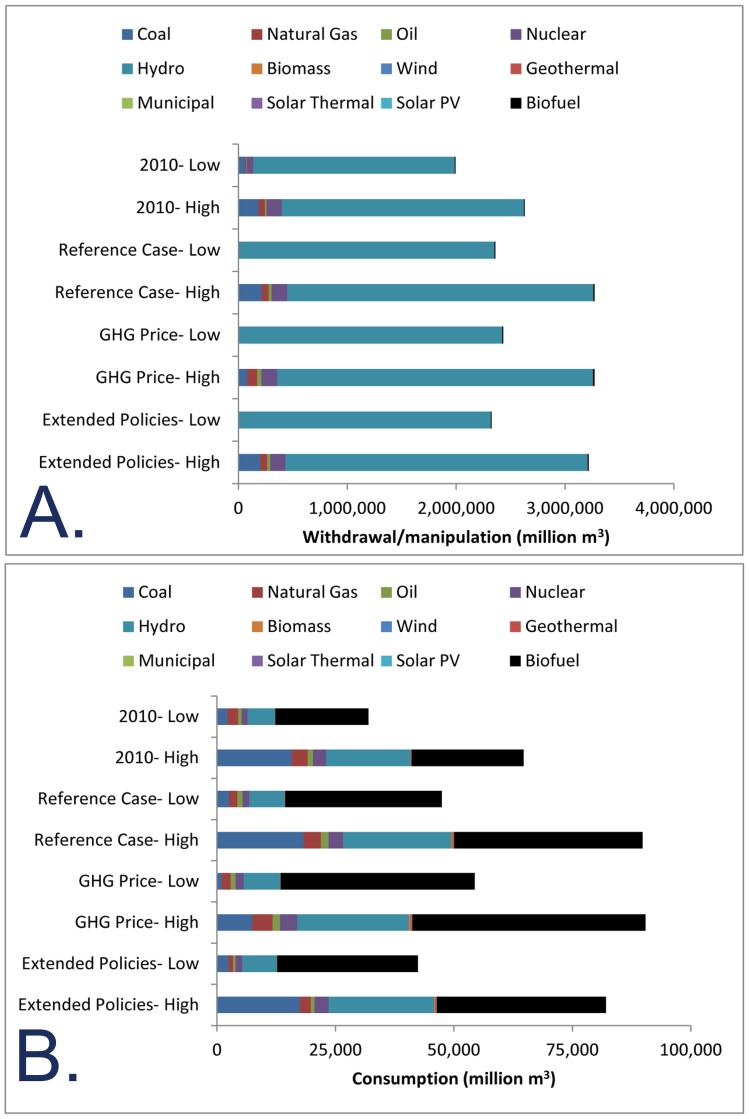
Water use with different energy policies. Water withdrawn (A) and consumed (B) for US domestic energy production, in Mm^3^, in 2010 and in 2035 for three scenarios of future energy policy. For each scenario, we show the value implied by our low and high water-use intensity estimates (Figure 3). Note the different scales between the two graphs.

Hydropower currently accounts for the largest total withdrawal/manipulation (1,851,000–2,222,000 Mm^3^) by far (Figure 4A), followed by coal (63,500–186,000 Mm^3^) and nuclear (58,200–140,000 Mm^3^). Note that for future scenarios our high-intensity number assumes a slow shift in technology as new thermoelectric plants use recirculating cooling and old plants gradually cease operations. By contrast, our low-intensity number describes a future in which EPA regulations or incentives drive all older plants to use recirculating cooling by 2035. By 2035, the Reference Case predicts that overall water withdrawal/manipulation will increase, because of additional hydroelectric, biofuel, and nuclear energy production.

The rank ordering of energy technologies is different with regards to current water consumption (Figure 4B), led by hydropower (5,830–17,800 Mm^3^), and followed by biofuels (4,300–5,300 Mm^3^), coal (2,240–15,800 Mm^3^) and natural gas (2,240–3,410 Mm^3^). Any shift toward a greater fraction of thermoelectric plants using recirculating cooling, rather than once-through cooling, does not greatly affect total consumption, since consumption water-use intensities for the two technologies are similar. The GHG Price Case predicts similar water consumption to the Reference Case, while the Extended Policies case predicts less water consumption due to decreased energy consumption.

**Figure 5:**
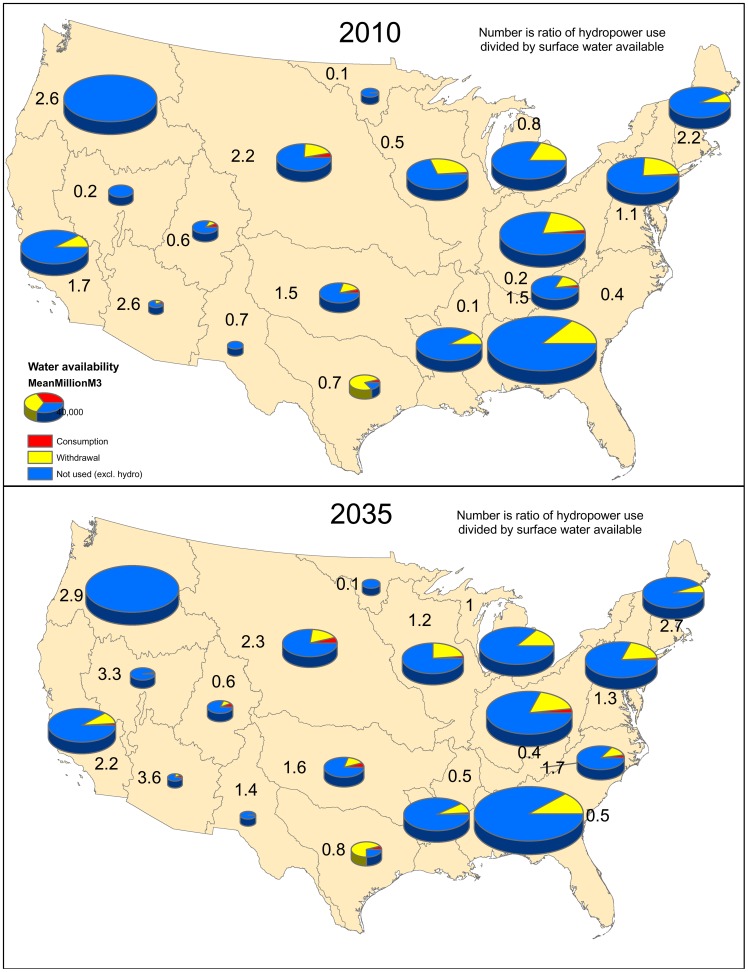
Water use by major water resource regions. Water use by the energy sector in major water resource regions in 2010 (A) and 2035 (B), under the Reference Case. The size of the pie chart indicates the total water available (mean Mm^3^ per year) in major water resource regions. The pie chart is divided into three colors, based on energy sector water use (excluding hydropower production). Water not used by the energy sector is shown in blue, while water withdrawn but not consumed is shown in yellow, and water withdrawn and consumed is shown in red. Then, the number in each region indicates the amount of water used specifically for hydropower production divided by total water available.

National-level statistics of energy sector water use mask significant variation among major water use regions (Figure 5A). The largest hydroelectric water withdrawal/manipulation, relative to the average annual water availability, is in the New England, Missouri, Lower Colorado, and Pacific Northwest regions, where the average water molecule has gone through more than two hydroelectric turbines by the time it flows to the ocean. By contrast, hydropower withdrawal/manipulation are a small fraction of the available water in the Great Basin, Ohio, Lower Mississippi, and Souris-Red-Rainy regions. Estimated hydroelectric water withdrawal/manipulation in million m^3^ is shown in Table 4. Under the Reference Case, some water resource regions such as the Great Basin, Lower Colorado, Upper Mississippi, and Rio Grande regions are projected to have a substantial increase in hydroelectric power (Figure 5B). The other two scenarios predict similar spatial patterns as the Reference Case.

**Table 4:** Water withdrawal/manipulation and consumption by major hydrologic region.

	Withdrawal/manipulation (million m^3^)			Consumption (million m^3^)		
Water Resource Region	*Thermoelectric*	*Hydropower*	*Biomass production*	*Thermoelectric*	*Hydropower*	*Biomass production*
New England	4,340-11,216	213,207–255,848	0	116–310	96–294	0
Mid-Atlantic	17,237-46,934	140,466–168,559	12–17	635–1,783	172–525	11–14
South Atlantic-Gulf	23,497-60,122	123,343–148,011	57–69	789–2,604	423–1,294	49–56
Great Lakes	14,512–38,488	116,730–140,076	28–38	403–1,340	222–678	25–31
Ohio	20,255–57,933	45,794–54,953	16–22	1,163–4,797	235–717	14–20
Tennessee	5,981–14,830	84,374–101,249	3	162–450	1,101–3,366	3
Upper Mississippi	15,895–42,835	47,648–57,178	18–26	441–1,905	22–67	17–23
Lower Mississippi	4,745–17,421	10,601–12,722	71–102	164–958	57–175	61–81
Souris-Red-Rainy	48–127	1,128–354	10–13	6	1	8–11
Missouri	7,752–26,302	165,476–198,572	1,331–1,716	351–2,557	139–426	1,156–1,395
Arkansas-White-Red	3,709–12,868	63,121–75,745	213–270	219–1,493	690–2,110	184–219
Texas-Gulf	8,936–31,733	17,192–20,630	124–156	327–1,569	78–237	108–127
Rio Grande	3–29	4,613–5,536	46–63	8	30–91	40–51
Upper Colorado	295–5,402	10,127–12,153	100–141	161–1,617	238–26	86–114
Lower Colorado	855–2,353	14,333–17,199	9–13	52–209	510–1,558	8–10
Great Basin	33–228	3,696–4,436	18–26	18–84	10–30	15–21
Pacific Northwest	806–2,419	587,454–04,944	146–182	90–292	986–3,016	127–148
California	5,129–19,232	202,466–42,959	63–82	214–504	818–2,502	55–67

As a share of available water, the largest withdrawal (excluding hydropower use) is in the Texas-Gulf and Lower Colorado water resource regions (Figure 5A). The lowest withdrawal relative to availability is in the Pacific Northwest, Great Basin, and Souris-Red-Rainy water resource regions. The vast majority of all withdrawals (excluding hydropower use) are for thermoelectric plant cooling, although the Missouri River basin also has a significant amount of water withdrawn for irrigation for bioenergy production (Table 4). By 2035, the Reference Case predicts an increase in energy sector withdrawal (Figure 5B), driven by irrigation water for energy crops, particularly corn for ethanol production. At the same time, there may be a decrease in withdrawals by 2035 in some regions like the Texas-Gulf and Lower Colorado water resource regions, depending on the rate of transition from once-through to recirculating cooling.

Relative to available water, consumption by the energy sector is generally low (Figure 5A). The sources of water consumption vary significantly among water resource regions (Table 4). In the majority of water resource regions, consumption by thermoelectric power plants is the dominant source of consumption. However, in a few water resource regions, like the Tennessee and the Lower Colorado, water consumed by hydropower (i.e., evaporation off reservoirs) is the dominant source of water consumption. Water consumed in bioenergy production is a significant part of total energy sector water consumption in the Missouri water resource region. By 2035, the Reference Case predicts an increase in biofuel water consumption (Figure 5B), primarily in the Missouri and Upper Mississippi water resource regions, and an increase in hydroelectric consumption, which is largest in the Great Basin and Lower Colorado water resource regions.

### Implications for fish species

Our metric of greater normalized hydroelectric water manipulation (i.e., hydropower use through turbines divided by water availability) appears consistent with expert evaluation of the threat dams pose to fish species. Fish for which “dams/impoundments” were reported as a threat had an average normalized hydroelectric water manipulation of 0.85, whereas those fish species where it was not reported as a threat had an average normalized hydroelectric water manipulation of 0.76.

**Figure 6:**
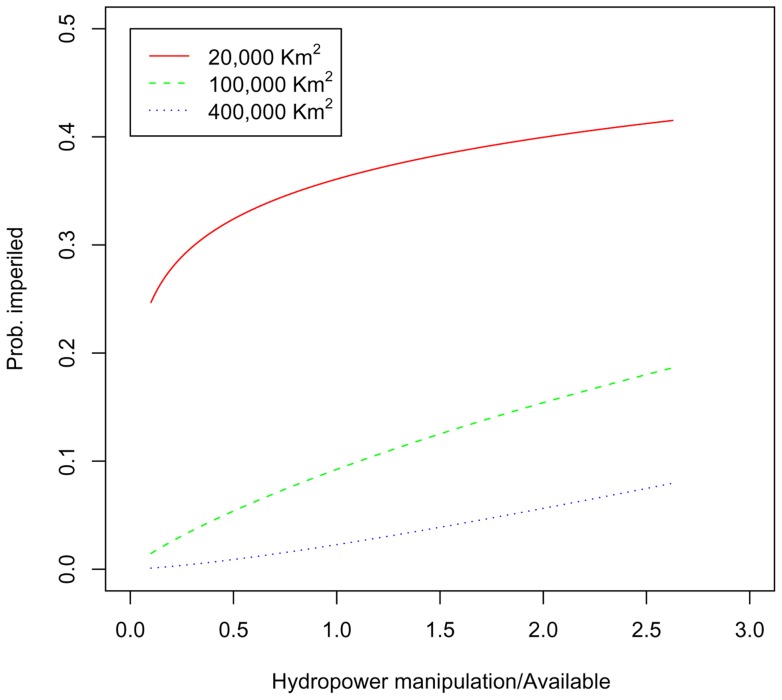
Fish imperilment as a function of hydropower water manipulation. Probability of a fish species being imperiled, as a function of the normalized hydropower water manipulation (i.e., water used in turbines/available). Curves are shown for three range sizes (km^2^), corresponding to the 25, 50, and 75^th^ percentile of fish species range sizes.

Water resource regions with greater normalized hydroelectric water manipulation have a greater proportion of imperiled fish species, after controlling for species range size and its interaction with normalized hydroelectric water manipulation (χ^2^ = 255.97, df = 3, P<0.001, Table 5). The effect of the interaction term in this best-fit model can best be seen graphically (Figure 6). Species with smaller ranges have a higher probability of being imperiled at any level of normalized hydroelectric water manipulation than do species with larger ranges. Moreover, the probability of imperilment for species with small ranges increase more rapidly with increases in normalized hydroelectric water manipulation than do species with large ranges.

**Table 5:** Probability of a fish species being imperiled as a function of normalized hydropower manipulation.

Predictor	β	SE β	Wald's χ^2^	df	P	*e* ^β^ (odds-ratio)
Intercept	9.97	0.97	104.92	1	<0.001	NA
LN(Normalized hydropower water manipulation)	−3.47	1.23	7.94	1	0.0048	0.031
LN(Total Range)	−1.06	0.095	126.32	1	<0.001	0.347
Interaction	0.37	0.12	9.05	1	0.0026	1.45
**Test**			**χ^2^**	**df**	**P**	
Overall model evaluation:						
Likelihood ratio test			255.97	3	<0.001	
Score test			210.63	3	<0.001	
Wald test			133.17	3	<0.001	

Note: Kendall's Tau-*a* = 0.240. Goodman-Kruskal Gamma  = 0.763. Somers's *Dxy*  = 0.762, *c*-statistic  = 88.1%.

Similarly, our metric of greater normalized energy sector water consumption (i.e., consumption divided by water availability) appears consistent with expert evaluation of the threat posed to fish species. Fish for which “excess withdrawals/consumption” were reported as a threat had an average normalized energy sector water consumption of 0.011, whereas those fish species where it was not reported as a threat had an average normalized energy sector water consumption of 0.008.

**Figure 7:**
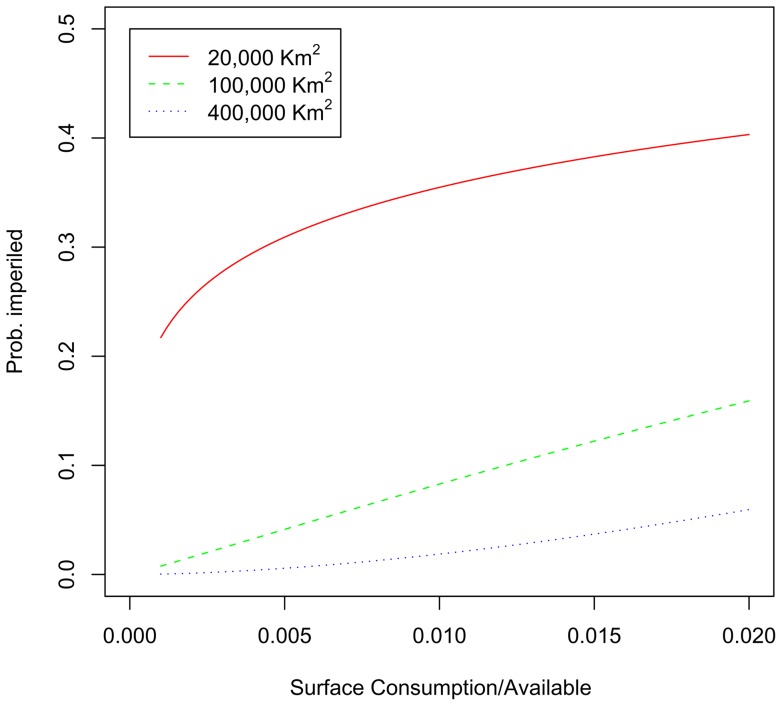
Fish imperilment as a function of surface water consumption. Probability of a fish species being imperiled, as a function of the normalized energy sector surface water consumption (i.e., consumption/available). Curves are shown for three range sizes (km^2^), corresponding to the 25, 50, and 75^th^ percentile of fish species range sizes.

Water resource regions with greater normalized energy sector water consumption have a greater proportion of imperiled fish species, after controlling for species range size and its interaction with normalized energy sector water consumption (χ^2^ = 268.78, df = 3, P<0.001, Table 6). The effect of the interaction term in this best-fit model can best be seen graphically (Figure 7). Species with smaller ranges have a higher probability of being imperiled at any level of normalized energy sector water consumption than do species with larger ranges. Moreover, species with small ranges increase more rapidly with increases in normalized energy sector water consumption than do species with large ranges.

**Table 6:** Probability of a fish species being imperiled as a function of normalized energy sector water consumption.

Predictor	β	SE β	Wald's χ^2^	df	P	*e* ^β^ (odds-ratio)
Intercept	−9.92	4.75	4.37	1	0.036	NA
LN(Normalized energy sector surface water consumption)	−4.44	1.07	17.30	1	<0.001	0.012
LN(Total Range)	1.08	0.47	5.20	1	0.023	2.94
Interaction	0.48	0.11	19.92	1	<0.001	1.6
**Test**			**χ^2^**	**df**	**P**	
Overall model evaluation:						
Likelihood ratio test			268.78	3	<0.001	
Score test			221.34	3	<0.001	
Wald test			128.97	3	<0.001	

Note: Kendall's Tau-*a* = 0.246. Goodman-Kruskal Gamma  = 0.784. Somers’s *Dxy*  = 0.783, *c*-statistic  = 89.1%.

Care must be taken when extrapolating these correlative patterns to the future, because our regression model was fit to cross-sectional data not panel data. However, some insight into which types of species are most likely to be imperiled in the future can be gained by examining a projection of regression results. In general, species with small ranges that are in water resource regions with a projected increase in energy-sector water consumption are most at risk. Under the Reference Case, water resource regions with an increase in consumption include the Lower Mississippi, the Texas-Gulf, and the Lower Colorado. Note that a more realistic evaluation of the effect of new energy sector water use on a particular fish species would require an analysis of the new energy development within that species range, as well as knowledge of the species-specific sensitivity to alterations to hydrological conditions associated with energy sector water use.

## Discussion

Our study revealed strong correlative relationships between current energy-sector water use and the likelihood of fish species imperilment in the US. If this association holds into the future then we expect that projected increases in energy-sector water use have the potential to further threaten freshwater fish. Since federal and state energy policies affect the combination of technologies used to produce energy, which in turn affects the amount of water withdrawn and consumed for energy production, energy policy decisions plays an important role in the conservation of freshwater fish biodiversity into the future. Below, we discuss a few key aspects of energy policy that affect how much energy sector water use occurs, and then explore their implications for fish species imperilment.

### Energy conservation

Policies that limit total energy consumption, either through increased energy efficiency or incentives to reduce energy use, conserve water. Energy efficiency gains are greatest in the Extended Policies Case, where strict energy efficiency standards for buildings and appliances and tighter Corporate Average Fuel Economy (CAFE) standards would reduce energy production by 5,000 PJ below the Reference Case. Our calculations suggest this would save 33,900–54,300 Mm^3^ of annual withdrawal/manipulation (2,510–4,650 Mm^3^ of consumption). In general, policies that limit use of liquid fuels would cause a slightly greater reduction in water consumption per unit energy (m^3^GJ^−1^) than would policies that limit electricity use, largely because the consumptive water-use intensity of corn ethanol production is so high.

### Climate policy

The effect of climate policy on energy-sector water use is complicated and varies by energy-production technology. The GHG Price Case would reduce total energy production by 4,750 PJ below the Reference Case, as higher electricity prices from fossil fuel sources would drive reductions in energy use. The GHG Price Case would also reduce water use by accelerating the retirement of old thermoelectric plants that disproportionately use once-through cooling. It would also shift some electricity production to renewable technologies that have relatively low water-use intensities. This would reduce withdrawals by thermoelectric plants by 1,740–91,000 Mm^3^ (1,180–9,000 Mm^3^ of consumption). Our study did not consider the effect of CCS technology on water use. Chandell et al. [13] estimated that CCS could increase water-use intensity by 25%, but their results are in agreement with our finding that overall climate policy would decrease water use by thermoelectric plants. However, the effects of climate policy on water-use in the liquid fuels sector are less clear. Increased biofuel production under the GHG Price Case means that withdrawals and consumption for this sector would increase by an even greater amount than the savings in the thermoelectric sector. The net effect is that the GHG Price Case withdrawal/manipulation would be approximately equal in 2035 to those in the Reference Case, as decreased withdrawals by thermoelectric plants are offset by increased biofuel production.

### Bioenergy production

All of our scenarios assume full implementation of the strong incentives for biofuel production mandated by the Energy Independence and Security Act of 2007 (EISA). Increased biofuel from corn and soybeans will increase the amount of water used for energy in the US, since both crops are occasionally irrigated. Note that our analysis assumed that new feedstocks for cellulosic ethanol are entirely rain-fed. If, however, feedstocks for cellulosic ethanol require irrigation, at least in some places in the US, then the water required for biofuel production may increase significantly.

### Thermoelectric plant cooling

The switch of thermoelectric plants from once-through cooling to recirculating cooling could substantially reduce water withdrawals. The biggest unknown here is how fast this shift will be, which is a function of market dynamics (i.e., how fast existing once-through cooled plants are retired) and policy regulation (i.e., if EPA regulations encourage existing plants to convert to recirculating cooling). Our calculations show that, in the Reference Case, conversion of all existing plants to recirculating cooling over a 20-year period would reduce annual water withdrawals in 2035 by 441,000 Mm^3^, relative to a continuation of the current gradual shift as existing once-through cooling plants are retired and new recirculating cooling ones come online. However, the switch from once-through cooling to recirculating cooling would not significantly reduce water consumption, which appears to have a greater impact, and is more predictive of native fish imperilment compared to water withdrawal. Moreover, the greatest reductions in withdrawals from the switch from once-through cooling to recirculating would be in the Northeast and Midwest, areas that have relatively low levels of fish endemism and imperilment.

### Implications for fish imperilment

The potential future impacts of energy-sector water use vary significantly by water resource region. Our statistical results show that fish species most likely to be imperiled have small ranges and are in water resource regions with high energy-sector normalized water consumption. The Southwestern US has both factors, with a large number of species with small ranges and high normalized water consumption, reinforcing the threat of water development on arid and semi-arid fish [37]. The Southeastern US also has high levels of species endemism and relatively high normalized water consumption. One implication of our results is that irrigation water for biofuel feedstocks may impact fish in places such as the Missouri and Arkansas-White-Red water resource regions. However, it is worth remembering that while our analysis looked at aggregate energy-sector consumption, different kinds of water consumption may have very different effects on fish. Water consumption in thermoelectric plant cooling likely has different ecological effects on particular fish species than water consumption in hydroelectric power consumption or during irrigation of bioenergy crops. More species-specific and basin-specific analyses are needed to understand the components of energy sector water consumption that are of most concern for species imperilment.

### Conclusions

Our results emphasize that policy decisions about energy are also decisions about water use, and that water sustainability and the health of freshwater ecosystems should be fully considered among the many factors that drive energy policy. The per-unit energy impacts of different energy technologies on water use, land use [26], and GHG emissions [38]–[41] vary dramatically. Energy policy involves complex tradeoffs among different aspects of freshwater ecosystems, economic needs, food security, climate change, and energy security. Nevertheless, certain technologies seem to have limited adverse impacts regardless of future scenarios. Energy efficiency, most notably, saves water and land [26] while reducing GHG emissions, resulting in a more sustainable future for biodiversity and energy production.
